# Exploring Technology Supporting Aging-in-Place Using an Equity Lens Through Focus Groups and World Café–Informed Research Agenda: Qualitative Study

**DOI:** 10.2196/71093

**Published:** 2025-07-30

**Authors:** Marianne Saragosa, Ibukun-Oluwa Omolade Abejirinde, Evan MacEachern, Michelle LA Nelson, Kristina M Kokorelias, Sidra Bharmal, Brina Ludwig Prout, Marian Mohamed

**Affiliations:** 1 Science of Care Institute, Sinai Health, 1 Bridgepoint Drive, Toronto, ON, M4M 2B5, Canada, 1 416-461-8252 ext 2895; 2 Institute of Health Policy, Management and Evaluation, Dalla Lana School of Public Health, University of Toronto, Toronto, ON, Canada; 3 Institute for Better Health, Trillium Health Centre, Mississauga, ON, Canada; 4 Research and Innovation Institute, Women's College Hospital, Toronto, ON, Canada; 5 Dalla Lana School of Public Health, University of Toronto, Toronto, ON, Canada; 6 Lunenfeld-Tanenbaum Research Institute, Sinai Health, Toronto, ON, Canada; 7 Department of Geriatric Medicine, Sinai Health System and University Health Network, Toronto, ON, Canada; 8 Department of Occupational Science and Occupational Therapy, Temerty Faculty of Medicine, Rehabilitation Sciences Institute, University of Toronto, Toronto, ON, Canada; 9 Rehabilitation Sciences Institute, Temerty Faculty of Medicine, University of Toronto, Toronto, ON, Canada; 10 Hennick Bridgepoint Hospital, Toronto, ON, Canada

**Keywords:** aging in place, community, research agenda, older adult, world cafe, technology, equity

## Abstract

**Background:**

Older adults prefer to age in their home or community of choice, which could include naturally occurring retirement communities (NORCs). As a place with a high density of older adults, NORCs could be sites where technology is leveraged to support independence and aging in the right place. However, there is limited research on how technology adoption and use occur in NORCs in ways that support older adults.

**Objective:**

This study aims to cocreate a research agenda on equity-informed technology considerations that help older adults live independently in NORCs.

**Methods:**

This is a 2-phase sequential qualitative descriptive study of 5 community-based focus groups and an in-person World Café event. We use the focus group method to acquire data about older adults’ experiences with and perceptions of using technology to support aging-in-place in NORC settings. This data informs the design and facilitation of deliberate dialogues at the World Café event. Three questions helped to guide the small group discussions. The World Café is a creative, collaborative, and conversation-generating method that aims to generate exchanges between people with different views on a particular topic.

**Results:**

In total, 45 NORC residents participated in a focus group about their experience and use of technology. The data revealed 3 central categories that highlight the perception of the use of technology to support the independence of participants in their homes and communities, its challenges, and areas to consider when deploying technology for helping older adults age in place. The subsequent World Café event included 40 participants and a combination of NORC residents, service providers, researchers, technology innovators, and policy makers. Insights drawn from the focus groups and World Café informed a 10-question research agenda about equity-informed technology principles that span accessible support, accessible interfaces, affordable and equitable access, available digital literacy training, accessible data, and accessible partnerships.

**Conclusions:**

Our study explores NORCs as potential environments for offering a transformative opportunity to address equity considerations for technology supporting aging in place. Our findings and research agenda highlight critical areas for consideration, including leveraging partnerships, integrating public and private technology ecosystems, and designing technology with older users that evolves with the population’s needs. Notably, by embedding principles of equity, inclusivity, and user-centered design, the collective of developers, researchers, and service providers can ensure that emerging technology serves diverse aging populations equitably and effectively.

## Introduction

### Background

As the fastest-growing age demographic in Canada, older adults are expressing a strong desire to age in place [1]. This research defines an “older adult” as a person aged 65 years or older [2]. Aging in place refers to living safely, independently, and comfortably in one’s home and community regardless of age, income, or ability level [3]. However, “aging in the right place” has been considered a more appropriate goal, given that place often depends on an older person’s personal preference, circumstances, and care needs [4]. Advocates suggest expanding housing options for those unsuitable for long-term care by developing alternatives, such as senior apartments, assisted living residences, and so forth to create safer and more accessible living environments and support the desires of older adults to age in place [4,5]. Such settings could include adaptations made to the residential environments of older people for supporting their independence and improving their quality of life [6]. For example, incorporating digital technologies, such as movement sensors and medication dispensers, is increasingly used to mitigate declining cognitive and physical abilities [7].

One example of an alternative housing model is naturally occurring retirement communities (NORCs). Originally coined in 1986, NORCs are defined as a “housing development that is not planned or designed for older people, but which over time comes to house largely older people” [8]. The NORC Innovation Centre at University Health Network has expanded the conceptualization of NORCs to include that they may consist of communities designed to house many older adults but not purpose-built for this population in the way long-term care homes or retirement homes are [9]. NORCs can provide a model for healthy aging-in-place since many offer supportive service programs based on the needs of residents [8]. While the availability and capacity of government, social, and health service providers and policy makers can create opportunities for supportive programming [10], less is known about how technology is currently being leveraged within NORC environments to support the needs of residents. Technological advancements (eg, remote monitoring systems, wearable health devices, and mobile apps) have shown promise in supporting efforts to age in place [11,12]. For example, countries like the Netherlands and Spain have focused on technology to mitigate the need for additional care and promote independence and autonomy [13]. In particular, low-tech technologies are designed to be as simple as possible and can offer practical, low-cost, and accessible features [14]. Despite considerable public and private interest and investment in aging-in-place technology development and research [15,16], there has been limited adoption of advanced technologies and their impact on people and health care systems [17], including diverse older adults [18,19]. Gaps persist in accessing technology and enabling digital literacy to support aging in the place where one prefers [20].

Later in life, technology acceptance and adoption are often influenced by the digital divide, which is known as the gap between those who do and those who do not have access to or knowledge of how to use advanced forms of technology [21]. While many older adults actively use current technologies (eg, internet, tablets, and smartphones), the digital divide continues to be a barrier for older people with lower access (ie, devices, internet, financial, and digital literacy) [22]. The digital divide is even more pronounced in those who experience marginalization at the intersections of racial and ethnic identity and socioeconomic status [23-25]. Indeed, significant barriers to accessing or engaging with technologies for health-related needs could worsen racial and ethnic health disparities experienced by the aging population [25]. While technology can offer promising solutions to support older adults in Canada and other countries in aging in the right place [19], the digital divide, or the gap between those who do and those who do not have access to new digital and information technology, must be addressed [21].

### Objective

With limited research on how technology adoption and use occur in NORCs in ways that support aging in place, this study aimed to cocreate a research agenda on equity-informed technology considerations that help older adults live independently in NORCs.

## Methods

### Study Design

This 2-phase sequential qualitative descriptive study [26] consisted of community-based in-person focus groups and an intersectoral World Café event. We used the focus group method to gather data about older adults’ experiences with and perceptions of using technology to support aging-in-place in NORC settings. The focus group data, in combination with project team discussions, helped develop the overarching questions for the deliberate dialogues at the World Café event [27]. The World Café is a creative, collaborative, and conversation-generating method that aims to generate exchanges between people with different views on a particular topic [28].

Developed in 1995, the World Café is considered a participatory method [29] by shifting to a bottom-up approach by engaging as many different actors as possible in the data collection process [30]. The World Café was the preferred approach in this case, as it has been used extensively to convene community-dwelling older adults and intersectoral participants [31,32], including research prioritization activities [28]. The evidence also supports the World Café as a valuable tool for fostering productive conversations among individuals with lived experience. Finally, with World Cafés, a large and heterogeneous group of people can be brought together in a systematic and organized manner [30].

We used the following 5 steps of the World Café method to guide our event planning: creating an informal environment, offering a warm welcome and overview from the host, engaging in 3 rounds of conversations among smaller groups of participants on 3 key questions (see ), and a final round of sharing the conversation results with the broader group [29]. Typically, participants are encouraged to rotate tables intermittently and meet new people; however, since some participants had mobility differences, we adapted the format such that the “tabletop host” and a notetaker (ie, scribe) rotated tables instead. The following were the World Café questions:

1. What challenges do older adults living in naturally occurring retirement community (NORC) neighborhoods have that might be supported with technology to help them stay in their homes?
2. How might challenges with technology be the same or different for people who are newcomers, are from different cultural backgrounds, education, and income levels?
3. What other things should we think about when developing and using technological (or digital) tools that could help older people stay in their homes?

### Sample and Recruitment

#### Focus Groups

We used purposeful sampling to identify and invite older adults living in urban NORCs in Toronto, Canada, to participate in a focus group. The inclusion criteria were community-dwelling older adults living in a NORC building. We identified NORCs from a publicly available list, including identified apartments, condos, co-operative housing, and social housing buildings within a bounded geographical area [33]. We used personal and professional networks to approach NORC buildings about the study. With agreement and support from the condo board or service providers at each location, we invited residents through word-of-mouth, circulating a recruitment flyer and using gatekeepers (ie, NORC residents or managers who brokered relationships between the researchers and the NORC building).

#### World Café

We also used purposeful sampling to invite interested focus group participants to the World Café. To ensure adequate representation of NORC residents in numbers, we used snowball sampling, encouraging focus group participants to share information about the World Café event with other residents. Because of the World Café’s intersectoral nature, we invited representatives from housing, health and social care, academics and research, and government senior services to attend.

### Data Collection

#### Focus Groups

Focus groups were held on-site in communal activity rooms within 5 different NORC buildings between April 2024 and June 2024. Each focus group involved a facilitator and a cofacilitator who supported the consent process, collected sociodemographic information, took field notes, and provided follow-up questions. The facilitator started each session by welcoming the participants, explaining the discussion’s purpose, and inviting them to introduce themselves. With an interview guide, the facilitator guided the discussion to address the following three central questions: (1) tell me about your current care needs; (2) tell me how technology addresses or could address these needs; and (3) tell me about current challenges and benefits with technology. For the focus group participants, we broadly defined “technology” as the various tools, devices, and systems people use daily to facilitate tasks, improve efficiency, and enhance convenience, spanning assistive devices, telemedicine, health monitoring, information and communication technologies, and so forth [34]. The focus groups lasted approximately 45-70 minutes; the discussions were digitally audio-recorded and transcribed verbatim by one of the authors (MM).

#### World Café

The in-person, full-day World Café event occurred in June 2024 in a large workspace at a tertiary hospital in Toronto, Canada. Upon arrival, participants were asked to complete paperwork (eg, consent forms and demographic survey) and were seated at one of six tables. Each table had a tabletop host and a notetaker. The host and notetaker were assigned one of three questions and rotated at 3 intervals to allow all participants to build on the prior responses to the question and provide additional feedback. Upon completion of the third round, all participants were invited to participate in a group discussion, answering the final question: “What groups need to work together to ensure that new technologies that help older people stay in their homes treat everyone fairly?”

### Data Analysis

#### Focus Groups

We used conventional content analysis to identify cross-cutting categories across the focus groups [35]. Conventional content analysis is most beneficial in the absence of existing research on the phenomenon. Therefore, it uses inductive logic, avoiding applying any predetermined categorical interpretation system [35]. Data analysis started with reading all transcripts, and 2 coders identified salient codes by highlighting exact words from the text to capture thoughts or concepts that appeared to describe an experience or perception of technology within the context of aging in place. Next, the first author created labels that reflected more than one concept, thereby sorting and merging codes into categories. Finally, the emergent categories were used to organize and group all the codes into meaningful clusters. For example, quotes like “[using technology to] connect with people near and far” and “interacted with all the kids and grandkids” were coded as “communication” and categorized under “Uses of technology.”

#### World Café

For the World Café event, we used directed content analysis. This analysis seeks to validate or extend a theory or framework [35]. In this case, we applied the Social Ecological Model (SEM) [36] as an organizing framework to map the factors of technology use among NORC residents, considering the challenges and enablers. The SEM was selected because it suggests that behavior is integrated into a dynamic network of intrapersonal characteristics, interpersonal processes, and institutional, community, and public policy factors [37]. Using the SEM also allows for identifying specific drivers of technology use or disuse among community members. Handwritten notes were transcribed and consolidated into a Microsoft Word document. In the coding process, we included examples for each of the factors. For instance, older adults’ knowledge, attitudes, and behaviors are at the “individual” level of the SEM model. Similar to the focus group analysis, we reviewed the transcribed notes and coded the data using predetermined categories wherever possible. For example, better integrating “sensors in NORC buildings” and coordinating specialized technology training, as a NORC was coded according to the SEM’s community level, acknowledges these factors as potential facilitators of aging-in-place. We also coded data that could not be categorized into one of the SEM factors, such as data related to technology devices and their features, design, and interface.

### Ethical Considerations

This study was approved by the Mount Sinai Hospital Research Ethics Board (REB reference number 23‐0123-E). Study participants provided written informed consent before engaging in study activities. Three participants in each focus group received a $25 CAD (US $18) gift card as a draw, whereas World Café attendees were offered a $25 CAD (US $18) gift card with the option to decline. Participation was not anonymous due to the focus group and in-person event; however, participation was voluntary. The study findings will be disseminated through presentations at conferences and peer-reviewed publications using deidentified data.

## Results

### Characteristics of Participants

#### Focus Groups

In total, 45 people participated in a focus group, with each focus group having between 6 and 25 NORC residents. Of the demographic data we collected, most of the participants identified as female (n=30, 67%), between 70 and 89 years of age (n=33, 74%). The most identified ethnicities were White North American or European (n=29, 66%), Southeast Asian (n=3, 7%), and East Asian (n=3, 7%). Most participants indicated living alone (n=27, 60%), followed by living with a partner (n=14, 31%). The remaining participants lived with others (including family) or preferred to keep their information private. See Table 1 for Focus group participant characteristics.

**Table 1.** Focus group participant characteristics.

Characteristic	NORC^a^ resident (n=45)
Gender, n (%)	
Women	30 (67)
Men	15 (33)
Age (y), n (%)	
60‐69	10 (22)
70‐79	21 (47)
80‐89	12 (27)
90‐99	1 (2)
Prefer not to answer	1 (2)
Ethnicity, n (%)	
White Northern American	19 (42)
White European	10 (22)
East Asian	3 (7)
Southeast Asian	3 (7)
Latino or Hispanic	2 (4)
Caribbean	1 (2)
Black African	1 (2)
Middle Eastern	1 (2)
Mixed heritage	1 (2)
Indigenous	1 (2)
Prefer not to answer	3 (7)
Living situation, n (%)	
Live alone	27 (60)
Live with partner	14 (31)
Live with family or friends	2 (4)
Prefer not to answer	2 (4)
Relationship status, n (%)	
Single	22 (49)
In a relationship	13 (29)
Widow	3 (7)
Separated	2 (4)
Prefer not to answer	5 (11)
Employment status, n (%)	
Retired	37 (82)
Unemployed, looking for work	3 (7)
Employed for wages	1 (2)
Prefer not to answer	4 (9)

a NORC: naturally occurring retirement community.

#### World Café

A total of 40 people participated in the World Cafe. Of these, 18 identified themselves as NORC residents; the remaining individuals were system leaders, including service providers, decision-makers, researchers, technology entrepreneurs, innovators, and administrators. Of the NORC residents, most identified as female (n=14, 78%), were between the ages of 70 and 79 (n=9, 50%) years, lived alone (n=9, 50%) or with a partner or family (n=8, 44%), and most identified as White European or North American (n=12, 66%). For the system leaders (N=22), the average years of experience was 11 (SD 4.2) years, ranging from 2 to 17 years. Most participants held a graduate degree (n=15, 68%) and identified as female (n=18, 82%). See Table 2 for World Café participant characteristics.

**Table 2.** World Café participant characteristics.

Characteristics	Role	
	NORC^a^ resident (n=18)	System leader (n=22)
Gender, n (%)		
Female	14 (78)	18 (82)
Male	4 (22)	4 (18)
Age (y), n (%)		
18‐29	0 (0)	1 (4)
30‐39	0 (0)	7 (32)
40‐49	0 (0)	11 (50)
50‐59	0 (0)	1 (4)
60‐69	4 (22)	0 (0)
70‐79	9 (50)	1 (4)
80‐89	4 (22)	0 (0)
90‐99	1 (6)	0 (0)
Prefer not to answer	0 (0)	1 (4)
Ethnicity, n (%)		
White European	8	5 (23)
White North American	4 (22)	5 (23)
East Asian	1 (6)	3 (14)
Southeast Asian	1 (6)	1 (4)
Caribbean	2 (11)	1 (4)
Black African	0 (0)	1 (4)
Middle Eastern	0 (0)	1 (4)
Mixed heritage	0 (0)	2 (9)
Prefer not to answer	2 (11)	3 (14)
Living situation, n (%)		
Live alone	9 (50)	—^b^
Live with partner	8	—
Live with family	1 (6)	—
Relationship status, n (%)		
In a relationship	8	—
Single	6 (33)	—
Widow	1 (6)	—
Prefer not to answer	3 (17)	—
Employment status, n (%)		
Retired	15 (83)	—
Unable to work	1 (6)	—
Self-employed	1 (6)	—
Prefer not to answer	1 (6)	—
Years of experience		
Mean (SD)	—	10.8 (4.2)
Range	—	2‐17
Profession, n (%)		
Researcher		11(50)
Service provider	—	4 (18)
Designer/innovator	—	4 (18)
Administrator	—	2 (9)
Policy maker	—	1 (5)
Education level completed, n (%)		
Postsecondary	—	6 (27)
Diploma	—	1 (5)
Graduate degree	—	15 (68)

a NORC: naturally occurring retirement community.

b Not applicable.

### Focus Group Findings

The focus group data revealed 3 central categories that highlight the use of technology to support participants’ independence in their homes and communities, its challenges, and areas to consider when deploying technology to help older adults age in place (see Table 3).

**Table 3.** Focus group findings.

Category	Open codes	Direct quotes
Using technology	Life is online Connecting everyday Finding information	“When I leave my appointment everything comes online…medical appointments, email, the whole world.” [FG 3] “If there’s something I don’t understand how to do, I Google it and it sends me to YouTube where it explains how to do it. But it’s so fast-paced that I have to keep rewinding it.” [FG 2]
Expressing challenges using technology	Goofing up the machine (cell phone) Remembering passwords	“I do not have a cell phone. I got rid of it a year and a half ago… I can goof the machine up very easy. I always seem to do that with cell phones.” [FG 2] “You have to remember to change your password every six months. We locked out and out because we forgot it.” [FG 4]
Identifying technology considerations	Needing to be taught Thinking about income	“My emails right now are coming into trash. And I’m at the point where I won’t touch anything.” [FG 2] “We have been waiting for the whole building to be so-called fiber. Well, unfortunately, they have been trying to entice me to join by asking me to spend $90.00 a month. For a retiree, it’s not really that simple” [FG 4]

#### Using Technology

Focus group participants described how they currently use technology to support their independence in their homes and communities. The types of use fell into categories of communication (eg, keeping in contact with family and friends), wellness and activity (eg, tracking upcoming appointments and online banking), information seeking (eg, accessing news, information, and current events), and for personal safety (eg, checking in on others). Some participants recognized the role of existing (eg, printer and video calling) and emerging technology (eg, ChatGPT and wearables) in adding value to their daily activities through access to information and connection to others.

*I have a new iPhone. And I wanted to use it to make films. So I figured to make a film, you have to have a story to start. So, I asked ChatGPT what story I could use. And it gave me a story that was much, much too complicated. So you have a conversation [with ChatGPT] about simplifying it and making it something you can do in two-minute films?*

[FG 1]

#### Expressing Challenges Using Technology

Cross-cutting challenges expressed by the participants included the cost associated with technology, such as data or internet plans or fall alert technology; physical changes that make using technology more complex; and concerns related to fraud and data privacy. The latter was expressed in all focus groups, highlighting the vulnerability felt by older adults to financial scams. Another challenge often mentioned was the need for more digital skills or the inability to use technology, such as cell phones, optimally. This challenge was well aligned with the expectation that older adults must become familiar with current technology, as many tasks related to day-to-day societal functioning have become digitalized. This includes accessing lab results or medical records and online banking. Several participants also disclosed individual physical and sensory changes that made navigating technology interfaces more difficult for them:

*I think some of us have physical problems. I have a lot of vision problems, so sometimes I can’t even do anything at all because I can’t see where the arrow is… the other thing is that I’m losing the ability to grasp with my hands, and that is horrible, but it, you know, it makes it difficult*

[FG 4]

#### Identifying Technology Considerations

Considering the challenges reported by our sample of NORC residents, many participants often suggested how to enhance their experience with technology. For example, they were intentional about receiving more technology support through education, training, and workshop-style learning. Our sample strongly desired someone to walk them through technology (eg, a digital navigator), especially when devices are seemingly not working and require troubleshooting advice. The desire for education also extended to avoiding cyber scams and using emerging technology, like ChatGPT. However, some participants resisted entirely internet-based systems and wondered about having either nontech or low-tech alternatives or technology that considered the unique needs of older adults, for example, a slower typing speed. Finally, participants also mentioned low-cost technology options aligned with their budgetary realities.

### World Café Findings

According to the workshop participants, the SEM framework helped highlight challenges and facilitators of technology at the individual, interprofessional, community, organizational, policy, and technology device levels (see Table 4).

**Table 4.** Social Ecological Model framework.

Level	Challenge	Facilitator
Individual (ie, knowledge, attitudes, and behaviors)	Fatigue with online appointments and expectation of online solutions (ie, “Zoom fatigue”) Hesitancy in asking for help with technology Need for repeated exploration to acquire and develop digital literacy (“One-and-done doesn’t work”) One’s formal education does not equate to digital literacy	Desire to have a “real” interaction Identified characteristics of those who adapt to technology: healthier, open-minded, able to afford technology-related costs, willingness to learn and acknowledge technology shortcomings
Interpersonal (ie, family, friends, and social networks)	Customer service supporting access to technology is not always respectful and informative Technology cannot replace human connection	Family available who can help facilitate set-up; family available to attend virtual meetings Free of charge or nominal cost-required services equipped to help older people stay connected (eg, Geek Squad and Toronto Public Library)
Community (ie, relationships between organizations)	Concerns about fraud and scams targeting older adults Virtual programming may result in challenges (ie, long waitlists, lack of relief to caregivers, or social participation for users) Ageism toward older people and technology, including them learning how to use technology	Technology to see what resources or assets are available in the community (eg, grocery delivery and volunteer services) Power in organizing a “collective idea or request” that supports collective learning and support for integrating technology solutions into living environments Having people in your building or community who can help and whom you can trust Building community and technology support (“economies of scale”), which may foster social engagement and participation Leverage existing, trusted institutions like public libraries
Organizational (ie, organizations and social institutions)	Customer service or technology support lacks the skills to communicate effectively with older adults (eg, they speak too loudly and give unclear instructions); people would rather live with the problem than deal with technology support	Technology tutorials or programs (eg, how to sign a document) Assistive devices in the home (eg, light sensors, passive sensors, and cane sensors) Existing support system (eg, NORC^a^ community coordinator) Creation of a “digital navigator” to facilitate solution finding and increase user participation
Policy (ie, national, provincial, and local laws)	Lack of policy requiring technology designed and customized to the needs of older users who consider sensory or cognitive differences The government seemingly not engaged in supporting use of technology among older adults	Affordable NORC phone, cell phone, and internet packages Having technology partners represented at the policy level
Technology device (ie, features, interface, and design)	Design does not meet the user needs, who may include older adults Language translation, colors, and fonts need to be unavailable or easily customizable for everyone (eg, bigger font and different colors)	Use of plain language or avoidance of acronyms Considering aging-related changes in technology design (eg, mouse speed and color contrast for vision)

a NORC: naturally occurring retirement community.

The workshop discussions revealed that technology is not generally tailored to meet the needs of older adults, and the aging-oriented devices that do exist can be improved through more thoughtful and unique designs. According to the workshop participants, co-design is needed, where technology developers work with older adults from the onset of the conceptualization and design process. Technology should not only be adapted for older adults but also specifically for different demographics and complex needs (eg, hearing impairment). An example of an improvement would be if device applications included multilingual options. Diverse groups of older adults must be consulted in technology development. Therefore, accessibility was frequently discussed, stressing the need for equity in technology design and access. In addition, the lack of affordability of technologies posed a significant barrier to accessibility, significantly disadvantaging lower-income groups. Participants suggested possible improvements, including reduced internet plans or group packages for NORCs.

The widespread adoption of technology to replace traditional services is becoming the default and can complicate many processes for older adults (eg, banking). Instead, there should continue to be a nononline option in addition to technology. The widespread use of technology is often blamed for reducing meaningful human interaction, which is concerning because people naturally seek social connection. Conversely, other participants voiced that information and communication technology allows connecting with others more easily, like family abroad, especially during scenarios of restricted movement, as in the case of the COVID-19 pandemic. Communication technologies (ie, Zoom [Zoom Communications] and Facetime [Apple Inc]) benefit virtual care, where technology can increase access to health care professionals and reduce wait times. Many participants noted they required more support for digital literacy to be comfortable navigating certain technologies, presenting a need for a tutor or someone available to answer technology-related questions, echoing insights from the focus groups.

It was suggested that aging communities, such as NORCs, should partner with programs or community agencies to support older adults in being technologically proficient. Technological literacy is critical in providing NORC residents with user confidence and helps to reduce anxiety around possible scams or security breaches. It was also suggested that instead of being compelled to accept the terms and conditions, users should be able to dictate the information that technology platforms share and with whom. Concurrently, technology could also provide a safety net at home. Many participants already appreciated using technology in remote monitoring and risk identification. Therefore, the ultimate goal to keep in mind when developing technology is how it can provide essential support in maintaining the independence of older adults to age in their preferred environment, which also evolves over an individual’s lifetime.

Synthesizing insights drawn from the focus groups and World Café informed a research agenda (Table 5) of questions mapped to equity-informed technology principles.

**Table 5.** Research agenda.

Principles	Research question
Accessible support	How can the NORC^a^ environment support access and use of technology among older residents by leveraging existing and new partnerships (ie, City of Toronto, Toronto Public Library, and Connected Canadians)? How do we leverage public (eg, Remote Care Monitoring) and private technology (eg, Apple Watch) ecosystems to support NORC residents to age in place? What might be the most impactful ways in which technology could support community building and participation among NORC residents, local service providers, and agencies?
Accessible interfaces	What are the technology design principles that consider the evolving needs of older adults, and how can these be integrated into current and future applications?
Affordable and equitable access	How can technology developers be guided to commit to equity and adhere to it during the design and deployment phases of emerging technology? How can government enact equitable and inclusive policies and funding to support the adoption of senior-friendly technology (ie, AgeTech)? Or policies to support alternatives to technology?
Available digital literacy training	How do we create community-based co-located senior-friendly technology education and training opportunities to meet the diverse needs of aging adults? How can education and training offerings be targeted to certain populations, for example, older adults who are new to Canada, those who are multilingual, are underhoused, and persons who have complex care needs?
Accessible data	How do we measure the impact of technology on NORC residents, and find data-driven solutions?
Accessible partnerships	How do we create an intersectoral and collaborative network of partners to support NORC residents to access and use technology, and who should be part of the network?

a NORC: naturally occurring retirement community.

## Discussions

### Principal Findings

We conducted a 2-phase sequential qualitative descriptive study of community-based focus groups and a World Café event to develop a research agenda on older adults’ informed considerations for technology to support the aging-in-place of persons living in NORCs. We found several cross-cutting categories across study activities. Participants described using technology for various reasons, including connecting with others, wellness and activity, information seeking, and personal safety. However, our sample’s technology usage was also impacted by barriers, such as limited access to (or outdated) devices, the cost associated with technology and its use (eg, internet), and a lack of available training or support to help fully use available technology or address low digital literacy within the context of age-related changes (ie, sensory, cognitive, and functional) that could hinder ease of adoption. The final set of 10 potential research questions underscores the importance of creating opportunities for age-friendly technology education, training, and equity in design, including design principles that reflect the evolving needs of older adults. Finally, the questions also emphasize the need to leverage intersectoral partnerships for supporting NORC residents who inhabit a unique living situation that enables the delivery of support within an existing community of persons aging in place. Therefore, accessing and using technology can serve as a mechanism for community building among NORCs and service providers.

### Comparison With Prior Work

Our findings on the barriers to using technology among older NORC residents are similar to other research that found challenges across intrinsic (including individual physical and sensory changes) and extrinsic (including inexperience with technology and cost to access) factors [38]. However, some sociodemographic factors may significantly impact specific groups of older adults, like those on lower or fixed incomes, females, ethnic minority groups, those with lower education levels, and those who are older [39,40]. While our study did not examine the intersectional impact of race and sex on access to technology, other research suggests a significant disparity, with non-White older females having the least access to technology [40]. These findings speak to the ongoing digital divide, referring to the gap between those with access to technology and those without. According to the literature, significant dimensions of the digital divide include digital literacy, affordability (costs), equity-denied group-related content and services (culturally appropriate content, activities, programs, and services), and access (infrastructure) [41].

NORCs, which include buildings or neighborhoods with a high density of older people, are home to highly diverse and socially and medically complex aging demographics [42,43]. Recent studies have found that NORC residents are older, more female, have lower incomes, live with more chronic conditions, and access home and primary care more often than non-NORC residents [43]. Together, these results suggest that there are more significant health needs than those of the general aging population; however, their communal living environment is particularly well-suited for leveraging. In this way, having a large concentration of older adults allows one to address technology needs in the same way health and social programming is delivered in NORCs [44], mainly through partnerships and collective efforts [45]. While we did not collect NORC participants’ income levels, medical histories, or educational backgrounds, leveraging the collective power of a NORC environment to arrange, for example, technology training was mentioned during the World Café forum.

Despite the increased interest in equity-informed technology for older adults, limited research exists regarding NORC residents’ adoption of technology that supports aging-in-place. Our findings align with previous interventional technology research (eg, sensory-based passive remote monitoring and smart speakers) implemented in NORC-like environments with lower-income and diverse older adults [46,47]. In these examples, access to a “cultural navigator” supported technology adoption through close relationships and information sessions [46]. However, without high-touch support for technology, its use among older residents may be limited to essential functions [47]. Similarly, NORC residents in another study expressed frustration and fear with using technology to access virtual programming during COVID-19 [48].

### Strengths and Limitations

Our study uniquely positions NORCs and related collaborative relationships as one approach to consider when addressing the digital divide. Learnings from the NORC context could inform future digital health research, including design and implementation studies focusing more broadly on aging-in-place. The research agenda drawn from this study provides guiding questions that could help identify priority areas when considering equity-informed technology deployment and use in NORCs; they include viewing the opportunity to support aging in place with technology through various lenses. Finally, multiple data sources informed the agenda development, including a rapid review, a focus group, and a World Café. Despite these strengths, this study is not without limitations. For instance, the study data reflect a group of NORC residents and stakeholder participants. Our sample may not be generalized across other NORC settings, such as those in many rural and northern communities and across racial and ethnic minority and low-income older adults. In these settings, limited high-speed broadband internet continues to be problematic [49]. Likewise, since the target population in our study was NORC residents, certain strategies could be used to gather similar information from other population groups. As noted in the literature, partnering with community organizations, relevant clinicians, health service planners, and policy makers can leverage existing networks while establishing new relationships [28]. These networks not only serve to optimize participation among different community members, but they could also support ongoing initiatives catalyzed by the engagement. We also acknowledge that focus groups and World Café participants from NORCs had some degree of technology experience, which may indicate that we did not hear directly from those with minimal technical literacy. Therefore, a more targeted recruitment approach could have identified those significantly impacted by the digital divide. Another consideration is the potential influence that receiving the gift card could have had on participants’ decisions regarding participation, for instance, being unduly incentivized to join the World Café. Finally, our final research agenda did not undergo a consensus exercise with our study participants. Future research could involve finalizing the agenda through a more iterative process, asking participants to rank questions on impact and importance.

### Future Directions

Future research could tackle one or more of these questions by identifying conventional and nontraditional intersectoral partnerships between, for example, the public library system, academic health institutions, technology start-ups, the public education system, third-sector and charity organizations, and community-based service and housing providers. These new collaborative intersectoral models could help provide more accessible assistance to older adults within a communal environment who seek technology support [50]. Our study has begun to explore NORCs as unique environments that offer a transformative opportunity to address equity considerations in technology supporting aging in place.

### Conclusions

Our findings and research agenda highlight critical areas for consideration, including leveraging partnerships, integrating public and private technology ecosystems, and designing technology with older users that evolves with the population’s needs. Notably, by embedding principles of equity, inclusivity, and user-centered design, developers, researchers, and service providers can ensure that emerging technology serves diverse aging populations equitably and effectively.

## Abbreviations

NORC: naturally occurring retirement community
SEM: Social Ecological Model
